# *Sanghuangporus sanghuang* Mycelium Prevents Paracetamol-Induced Hepatotoxicity through Regulating the MAPK/NF-κB, Keap1/Nrf2/HO-1, TLR4/PI3K/Akt, and CaMKKβ/LKB1/AMPK Pathways and Suppressing Oxidative Stress and Inflammation

**DOI:** 10.3390/antiox10060897

**Published:** 2021-06-02

**Authors:** Wen-Ping Jiang, Jeng-Shyan Deng, Shyh-Shyun Huang, Sheng-Hua Wu, Chin-Chu Chen, Jung-Chun Liao, Hung-Yi Chen, Hui-Yi Lin, Guan-Jhong Huang

**Affiliations:** 1 Department of Chinese Pharmaceutical Sciences and Chinese Medicine Resources, College of Chinese Medicine, China Medical University, Taichung 404, Taiwan; wpjiang@gm.asia.edu.tw; 2 Department of Pharmacy, Chia Nan University of Pharmacy and Science, Tainan 717, Taiwan; 3 Department of Occupational Therapy, Asia University, Taichung 413, Taiwan; 4 Department of Health and Nutrition Biotechnology, Asia University, Taichung 413, Taiwan; dengjs@asia.edu.tw; 5 School of Pharmacy, China Medical University, Taichung 404, Taiwan; sshuang@mail.cmu.edu.tw (S.-S.H.); ljc@mail.cmu.edu.tw (J.-C.L.); hungyi@mail.cmu.edu.tw (H.-Y.C.); hylin@mail.cmu.edu.tw (H.-Y.L.); 6 Department of Biology, National Museum of Natural Science, Taichung 404, Taiwan; shwu@mail.nmns.edu.tw; 7 Grape King Biotechnology Center, Chung-Li City 320, Taiwan; gkbioeng@grapeking.com.tw

**Keywords:** *Sanghuangporus sanghuang*, paracetamol, hepatoprotective, MAPK/NF-κB pathway, Keap1/Nrf2/HO-1 pathway, CaMKKβ/LKB1/AMPK pathway, anti-inflammation

## Abstract

Liver damage induced by paracetamol overdose is the main cause of acute liver failure worldwide. In order to study the hepatoprotective effect of *Sanghuangporus sanghuang* mycelium (SS) on paracetamol-induced liver injury, SS was administered orally every day for 6 days in mice before paracetamol treatment. SS decreased serum aminotransferase activities and the lipid profiles, protecting against paracetamol hepatotoxicity in mice. Furthermore, SS inhibited the lipid peroxidation marker malondialdehyde (MDA), hepatic cytochrome P450 2E1 (CYP2E1), and the histopathological changes in the liver and decreased inflammatory activity by inhibiting the production of proinflammatory cytokines in paracetamol-induced acute liver failure. Moreover, SS improved the levels of glutathione (GSH), superoxide dismutase (SOD), glutathione peroxidase (GPx), and catalase in the liver. Significantly, SS diminished mitogen-activated protein kinase (MAPK), Toll-like receptor 4 (TLR4), phosphatidylinositol 3-kinase (PI3K)/protein kinase B (Akt), and the nuclear factor-kappa B (NF-κB) axis, as well as upregulated the Kelch-like ECH-associated protein 1 (Keap1)/erythroid 2-related factor 2 (Nrf2)/heme oxygenase-1 (HO-1) pathway, in paracetamol-induced mice. SS mainly inhibited the phosphorylation of the liver kinase B1 (LKB1), Ca^2+^/calmodulin-dependent kinase kinase β (CaMKKβ), and AMP-activated protein kinase (AMPK) protein expression. Furthermore, the protective effects of SS on paracetamol-induced hepatotoxicity were abolished by compound C, an AMPK inhibitor. In summary, we provide novel molecular evidence that SS protects liver cells from paracetamol-induced hepatotoxicity by inhibiting oxidative stress and inflammation.

## 1. Introduction

Paracetamol (N-acetyl-p-aminophenol), also known as acetaminophen, is the most common drug used to treat pain and fever, and is considered safe at the recommended therapeutic concentration. However, 150 mg/kg (or 12 g for the average person) is a toxic dose for adults and confers a high risk of liver damage, which may cause acute liver failure and even death. Paracetamol poisoning is clinically important because it accounts for 44% of the adult self-poisoning cases [1,2]. The toxicity induced by paracetamol is triggered by the formation of a metabolite, N-acetyl-p-benzoquinoneimine (NAPQI), which is catalyzed by cytochrome P450 CYP2E1, an enzyme whose excessive activity can cause liver damage by depleting glutathione (GSH) [3,4]. When GSH is depleted, the NAPQI formed reacts with cellular proteins and induces oxidative stress, leading to the necrosis of hepatocytes [4]. The resulting increase in superoxide production is essential for continuous pathological processes. The spontaneous reaction of superoxide and nitric oxide (NO) produces peroxynitrite, which plays an important role in the mechanism of paracetamol-induced liver toxicity.

Liver damage usually starts 24 to 72 h after a paracetamol overdose [5]. The clinical treatment of paracetamol-induced hepatotoxicity has its limitations. NAC (N-acetylcysteine) has become a standard treatment in the clinic. Although NAC displays great therapeutic potential in preventing paracetamol-induced acute liver failure, it must be administered as soon as possible after paracetamol overdose for it to exert its greatest effect. This may not be possible in most paracetamol overdose patients. Liver cell necrosis worsens with the decrease in antioxidant enzyme activity. It has been pointed out in the literature that exposure to excessive paracetamol in mice lacking the manganese superoxide dismutase (SOD2) gene can exacerbate liver damage [5,6]. Many compounds and extracts have been shown to have hepatoprotective activity, reducing paracetamol-induced liver injury through reducing reactive oxygen species (ROS), oxidative stress, and inflammatory mediators. Certain antioxidant enzymes (SOD, catalase and glutathione peroxidase (GPx)) are critically involved in the regulation of paracetamol-induced liver toxicity [7]. The main function of nuclear factor erythroid 2-related factor 2 (Nrf2) is regulating drug-metabolizing enzymes and antioxidant genes by binding to the antioxidant response elements (AREs) in their promoters, thereby reducing paracetamol’s hepatotoxic effects [8]. Kelch-like ECH-related protein 1 (Keap1) is the key negative regulator of Nrf2; the activation of the latter involves its release from Keap1, allowing it to induce the expression of numerous antioxidant and detoxification genes [9]. Heme oxygenase-1 (HO-1) is one such gene and has been shown to promote the lysis of heme, thereby accelerating the formation of biliverdin and reducing the production of intracellular ROS. The liver toxicity of paracetamol is mainly caused by oxidative stress. Because Nrf2 plays an important role in the defense against oxidative stress, the Keap1/Nrf2/HO-1 axis may help to protect against paracetamol-induced liver damage [10].

Nuclear factor-κB (NF-κB) regulates many genes involved in different processes of the immunomodulatory responses. The mechanism of NF-κB activation is the inducible degradation of IκBα triggered through its site-specific phosphorylation by a multi-subunit IκB kinase (IKK) complex. IKK can be evoked by various factors, including cytokines, growth factors, mitogens and stress agents [11]. The proinflammatory cytokine IL-6 plays an essential role in paracetamol-induced liver injury via Toll-like receptor (TLR) 4; TLR4 is directly involved in paracetamol-induced liver injury and inflammation [12]. Several studies have reported that the phosphatidylinositol 3-kinase/protein kinase B (PI3K/AKT) axis is associated with paracetamol-induced liver damage and early liver development and regeneration [13]. Based on these studies, we speculate that targeting the TLR4/PI3K/Akt/NF-κB axis could represent a new potential strategy for liver protection.

AMP-activated protein kinase (AMPK) is a serine/threonine protein kinase that serves as a key sensor of cellular energy status and is activated by an increase in the ratio of cellular AMP/ATP or ADP/ATP [14]. AMPK activation has been shown to inhibit inflammation in various model systems [15], such as by inhibiting the NF-κB axis, and enhance the antioxidant capacity of cells through inducing the nuclear localization of Nrf2 [16]. In addition, two upstream kinases, the liver kinase B1 (LKB1) and the Ca^2+^/calmodulin-dependent kinase kinase β (CaMKKβ), have been demonstrated to regulate AMPK. LKB1 regulates cellular signaling by redox homeostasis through the detoxification of ROS [17]. LKB1 also can regulate NF-kB-mediated macrophage activation. LKB1-deficient macrophages show higher production and expression of proinflammatory cytokines after lipopolysaccharide (LPS) stimulation and enhanced NF-kB activity [18]. Some reports indicate that CaMKKβ participates in NF-κB-mediated inflammatory signals, which may be involved in the proinflammatory activity of macrophages. Therefore, CaMKKβ/LKB1/AMPK may be involved in liver pathology, because inhibiting its activity can prevent the occurrence of liver diseases. The mechanism by which paracetamol causes liver damage is still unclear, but increasing evidence shows that mediators of oxidative stress and inflammation are involved.

*Sanghuangporus sanghuang* (SS)*,* a rare and precious medicinal fungus, has been used to treat several diseases in Taiwan, China, Japan, and Korea for hundreds of years [19,20]. In traditional usage, SS has been applied for the treatment of diarrhea, night sweats, hemorrhoids, eczema, stomach pain, bleeding, vaginal discharge, and amenorrhea. The pharmacological effects of SS are reported to include antioxidant, anti-inflammatory, anti-tumor, anti-aging, anti-diabetic, and antiviral effects [21,22,23]. The scientific name “*S. sanghuang*” was controversial before 2018; data then showed that *Phellinus linteus* or *Inonotus Sanghuang* was not the correct name and that it should be *S. sanghuang*, because a new species that only grew on live mulberry trees had been discovered. Data suggested that SS could efficiently block oxidative stress and inflammatory responses in paracetamol-induced liver injury and that its mechanism might be related to the MAPK/NF-κB, Keap1/Nrf2/HO-1, TLR4/PI3K/Akt, and CaMKKβ/LKB1/AMPK pathways. 

## 2. Materials and Methods

### 2.1. Reagents

Paracetamol, NAC, other chemicals and solvents were bought from Sigma-Aldrich (St. Louis, MO, USA). ELISA kits for detecting the release of mouse TNF-α, IL-1β, and IL-6 were purchased from BioLegend Inc. (San Diego, CA, USA). Primary antibodies for Western blotting against the proteins COX-2, p-JNK, catalase, GPx, SOD, CYP2E1, AMPK, LKB1, CaMKKβ, p-AMPK, p-LKB1, and p-CaMKKβ were purchased from GeneTex (San Antonio, TX, USA). Antibodies against JNK, p-ERK, ERK, p-p38, and p-IκB-α were purchased from Cell Signaling Technology (Beverly, MA, USA). Antibodies against iNOS, NF-κB, IκBα, p38, HO-1, Nrf-2, and β-actin were purchased from Abcam (Cambridge, UK, USA). Protein assay kits (Bio-Rad Laboratories Ltd., Watford, Herts, UK) were obtained as indicated.

### 2.2. Source of Material

The mycelium of *S. sanghuang* used in this study was fermented by Taiwan Grape King Biological Co., Ltd. (Chung-Li City, Taiwan), and its strain was proposed by Dr. Sheng-Hua Wu from the National Museum of Natural Science. The *S. sanghuang* mycelium originated on the trunk of Morus in Yanping Township, Taitung County, Taiwan. It was collected in 2011/5. The “voucher specimens” are stored in the National Museum of Natural Science (Wu 1105-1).

### 2.3. Sample Preparation

Dried mycelium powder was soaked in 70% ethanol for one week, and then, the residue was filtered out. The filtrate was concentrated under reduced pressure to remove the ethanol. This step was repeated three times to obtain the extract, which was then stored at −20 °C. 

### 2.4. Animals

Six- to seven-week old pathogen-free male ICR mice (body weights, 20–25 g) were obtained from BioLASCO Taiwan Co., Ltd. (Taipei, Taiwan). Six mice were maintained per cage in an animal room maintained at a constant temperature of 22 ± 1 °C, with a relative humidity between 50% and 60%, and light/dark cycle of 12/12 h, and were fed with a standard diet and water ad libitum. All the experimental procedures and methods have been approved by the Animal Management Committee of China Medical University (IACUC approval number: CMUIACUC-2016-376).

### 2.5. Experimental Protocol 

After about 1 week of adaptation, the male ICR mice were randomly assigned to six groups (*n* = 6 per group): the control group, paracetamol group, paracetamol + NAC (600 mg/kg) group, paracetamol + SS (125 mg/kg) group, paracetamol + SS (250 mg/kg) group, and paracetamol + SS (500 mg/kg) group. SS was suspended in 0.5% carboxymethyl cellulose (CMC) solution and orally administered to mice in three treatment groups at doses of 125, 250, and 500 mg/kg, respectively, for 6 days, with the last dose given 1 h before paracetamol administration. The control and paracetamol mice were only treated with 0.5% CMC solution, in the same way. One hour after the final SS dose, mice were administered with paracetamol (400 mg/kg) with normal saline by single intraperitoneal injections in all the groups (except the control group). The mice in the NAC group were orally pretreated with NAC (600 mg/kg) 1 h before the paracetamol challenge. Twelve hours after paracetamol administration, the mice were euthanized with CO_2_, and then, blood and liver tissues were collected for further analysis [24]. The blood was collected by cardiac puncture after euthanasia and centrifuged at 1700× *g* for 30 min at 4 °C for plasma collection.

To evaluate the role of an AMPK inhibitor (compound C) in regulating paracetamol-induced hepatotoxicity, mice were randomly divided into five groups (*n* = 6 per group): a control group, paracetamol (400 mg/kg) group, paracetamol (400 mg/kg) + compound C (25 mg/kg) group, paracetamol (400 mg/kg) + SS (500 mg/kg) group, and paracetamol (400 mg/kg) + SS (500 mg/kg) + compound C (25 mg/kg) group. SS was administered to mice for 6 days at doses of 500 mg/kg, respectively. The control and paracetamol mice were only treated with 0.5% CMC solution, in the same way. Compound C (25 mg/kg) was given intraperitoneally to the animals of the intervention groups 1 h prior to paracetamol administration. After fasting for 12 h, mice were intraperitoneally injected with paracetamol solution. Twelve hours after paracetamol administration, the mice were anesthetized for harvesting blood for further analysis. 

### 2.6. Analysis of Biochemical Markers

The blood was centrifuged (5 min at 12,000× *g* at 4 °C) to separate the serum. The serum levels of ALT (alanine aminotransferase), AST (aspartate aminotransferase), T-Bil (total bilirubin), TC (total cholesterol), and TG (triglyceride) were measured using commercial detection kits (HUMAN Diagnostics Worldwide, Ahrensburg, Germany). 

### 2.7. Histopathological Examination

The liver samples were fixed in 10% formalin for at least 24 h before paraffin embedding. The slides were stained with hematoxylin and eosin (H&E), and examined using a Nikon Compound Microscope (Nikon, ECLIPSE, TS100, Tokyo, Japan), to evaluate the cellular and morphological structure. The severity of liver disease was graded from 0 to 5: 0 points means normal (normal—no hepatocyte necrosis); 1 point means minimal–mild (less 1%) (focal and limited to centrilobular region; fewer than ¼ of the affected lobules are necrotic); 2 means mild–moderate (1–25%) (focal and multifocal central to midzonal lobular region; ½ of the affected lobules are necrotic); 3 means moderate–severe (26–50%) (multifocal (centrilobular–portal region); ½ to ¾ of the affected lobules are necrotic); 4 means severe (51–75%) (multifocal; over ¾ of the affected lobules are necrotic); 5 means severe (whole lobules) (76–100%) (hepatocyte loss from central vein to portal area extends to adjacent lobules) [25]. 

### 2.8. Analysis of MDA 

Excessive oxidative stress triggers lipid peroxidation, which leads to the formation of MDA. Thiobarbituric acid (TBA) reacts with MDA, which is estimated by the thiobarbituric acid reactive substances (TBARS). Liver tissue samples were combined with the TBA reagent, and the absorbance at 535 nm was measured. The concentration of TBARS was expressed as nmol/mg protein [26].

### 2.9. Analysis of GSH 

GSH concentrations were measured using DTNB (5,5-dithiobis (2-nitrobenzoic acid)). The samples were homogenized in ice-cold 10% TCA buffer and centrifuged at 3000 rpm for 10 min at 4 °C, and then, the supernatant was removed. The supernatant (0.1 mL) was mixed with 2.0 mL of phosphate buffer (0.3 M, pH 8.4), containing double-distilled water (0.4 mL) and DTNB (0.001 M, 0.5 mL). The optical density at 412 nm was read on a colorimeter. The measured absorbance values were compared with the standard curve generated using GSH of known concentrations. The concentration of GSH was expressed as µmol/g tissue [27].

### 2.10. Analysis of Serum NO, TNF-α, IL-1β, and IL-6

The nitrite level in the serum was indirectly assessed using Griess reagent (0.5% sulfanilamide and 0.05% N-1-naphthylethylenediamine). Briefly, serum was mixed with an equal volume of Griess reagent. After 10 min of incubation, the absorbance of the supernatants was measured using a microplate spectrophotometer at 540 nm [28]. According to the manufacturer’s instructions, the serum levels of proinflammatory factors (TNF-α, IL-1β, and IL-6) were evaluated using an enzyme-linked immunosorbent assay (ELISA) system. After the reaction, the absorbance was determined using an ELISA reader (Versa Max, Molecular Devices, CA, USA). The concentrations of the proinflammatory cytokines were expressed as pg/mL.

### 2.11. Western Blot Analysis

Liver tissue (30–50 mg) was collected and added to the RIPA buffer, and centrifuged at 12,000 rpm for 20 min at 4 °C. The proteins were extracted, diluted with loading buffer, loaded onto 10% SDS-PAGE gels in which they were electrophoresed, and then transferred to PVDF membranes (Millipore, Bedford, MA, USA). The membranes were then blocked with 5% non-fat milk for 1 h at room temperature and then washed and probed with corresponding primary antibodies and, subsequently, secondary antibodies. The protein bands were visualized using an ECL kit (Amersham International plc., Buckinghamshire, UK), and the density of the bands was analyzed using ImageJ (Bethesda, MD, USA).

### 2.12. Statistical Analysis

All the values are expressed as the mean ± standard error of the mean (S.E.M). Statistical analysis was performed using the one-way analysis of variance (ANOVA) or Student’s t-test for multiple comparisons. Differences between groups were considered to be statistically significant when *p* < 0.05 (SPSS version 20.0, SPSS Inc., IBM Corp., Armonk, NY, USA).

## 3. Results

### 3.1. The Preventive Effect of SS on Hepatocellular Damage 

**Figure 1:**
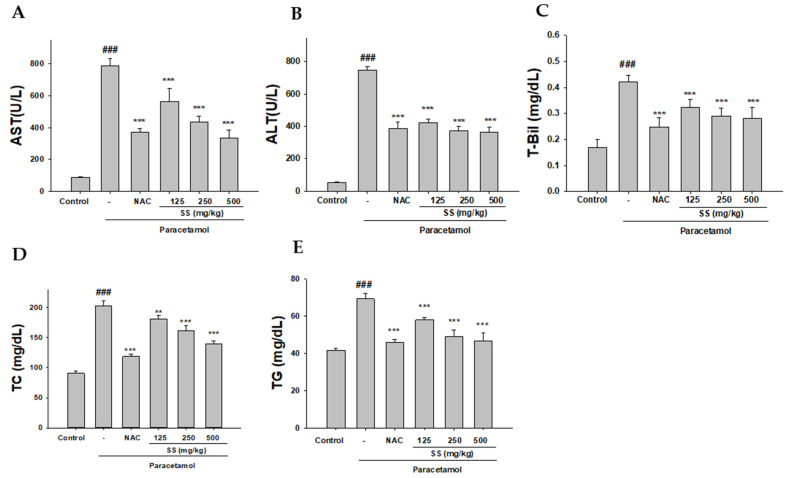
Effects of *S. sanghuang* mycelium (SS) on paracetamol-induced increase in serum AST (**A**), ALT (**B**), T-Bil (**C**), TC (**D**), and TG (**E**) levels. SS was orally administered to mice for 6 days, with the last dose 1 h before paracetamol administration. The values are reported as the means ± S.E.M. (*n* = 6). ^###^ *p* < 0.01 relative to control; ** *p* < 0.01 and *** *p* < 0.001 relative to the paracetamol group.

The levels of several plasma markers, including AST, ALT, and the lipid markers (T-Bil, TC and TG), were significantly increased in the paracetamol-treated group compared with the control group, confirming the hepatotoxicity of paracetamol overdose (Figure 1A–E). SS and NAC significantly inhibited the increase in the serum AST, ALT, and lipid markers; these results demonstrate that SS prevented the paracetamol-induced liver toxicity.

### 3.2. SS Alleviates Paracetamol Hepatotoxicity

**Figure 2:**
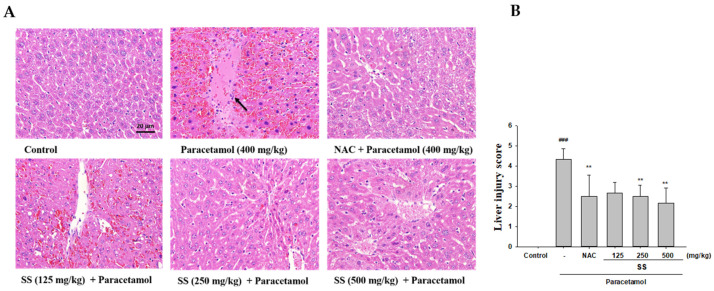
Effects of SS on histological alterations and the severity of liver injury in the liver after paracetamol overdose. Tissue sections were then stained with H&E (400x) (**A**) and the living injury score (**B**) and evaluated under a microscope. The data are presented as the means ± S.E.M (*n* = 6). ^###^ *p* < 0.01 relative to the control group. ** *p* < 0.01 relative to the paracetamol group. Arrowheads denote central veins and highlight liver injury/necrosis.

The analysis of the histopathological images shows that paracetamol toxicity is the leading cause of the morphological changes in the liver, including hepatic steatosis, inflammation in the hepatic lobules, the necrosis of centrilobular hepatocytes, and ballooned hepatocytes (Figure 2A). SS undoubtedly alleviates liver damage, and reduces liver cell necrosis and degeneration. In addition, the liver injury scores showed that SS could reduce inflammatory responses and resulted in a reduction in the necrosis grade compared to the paracetamol group (Figure 2B). Taken together, our histological results demonstrate that oral pretreatment with SS prevented the paracetamol toxicity.

### 3.3. Inhibition of Paracetamol-Induced Lipid Peroxidation and Preservation of the Levels of GSH by SS

**Figure 3:**
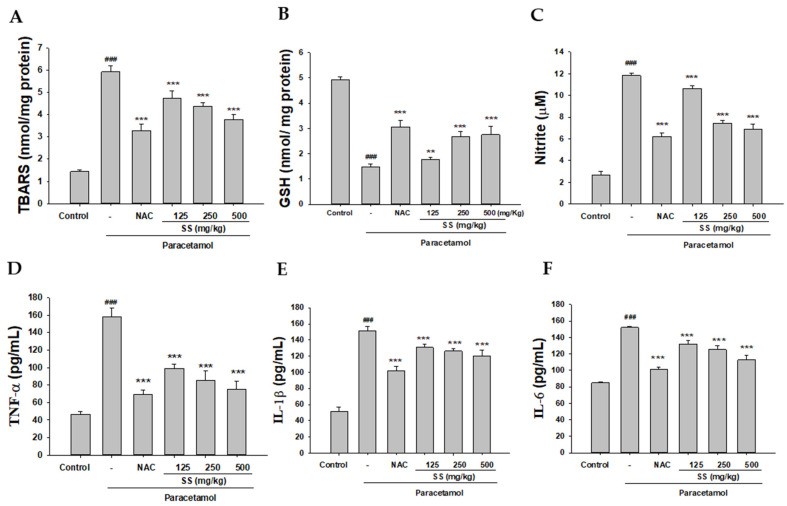
Pretreatment with SS decreased lipid peroxides (**A**), increased GSH (**B**) levels, and reduced levels of inflammatory cytokines NO (**C**), TNF-α (**D**), IL-1β (**E**), and IL-6 (**F**) in paracetamol-treated mice. SS was orally administered to mice for 6 days, with the last dose 1 h before paracetamol administration. GSH was determined and expressed as μmol/g liver tissue. Nitrite concentration in the serum was determined using Griess reagent. Serum concentrations of TNF-α, IL-1β, and IL-6 were determined using commercial ELISA kits. The values are reported as the means ± S.E.M. (*n* = 6) of five mice per group. ^###^ *p* < 0.01 relative to the control group. ** *p* < 0.01 and *** *p* < 0.001 relative to the paracetamol group.

The levels of TBARS were increased in the paracetamol group compared with the control group (Figure 3A). The pre-administration of SS markedly decreased the levels of TBARS compared with the paracetamol group. Our data confirm that the hepatoprotective effect of SS can be attributed to the antioxidant potential according to the reduction in lipid peroxidation.

Oxidative stress and inflammation are closely related to the pathogenesis of acute liver disease because the endogenous antioxidant system is often damaged, leading to severe tissue damage. As shown in Figure 3B, excessive paracetamol led to a significant decrease in GSH content in the liver compared with control. However, pretreatment with SS and NAC increased the GSH content compared with the paracetamol group.

### 3.4. Inhibition of Paracetamol-Induced Liver Inflammation

**Figure 4:**
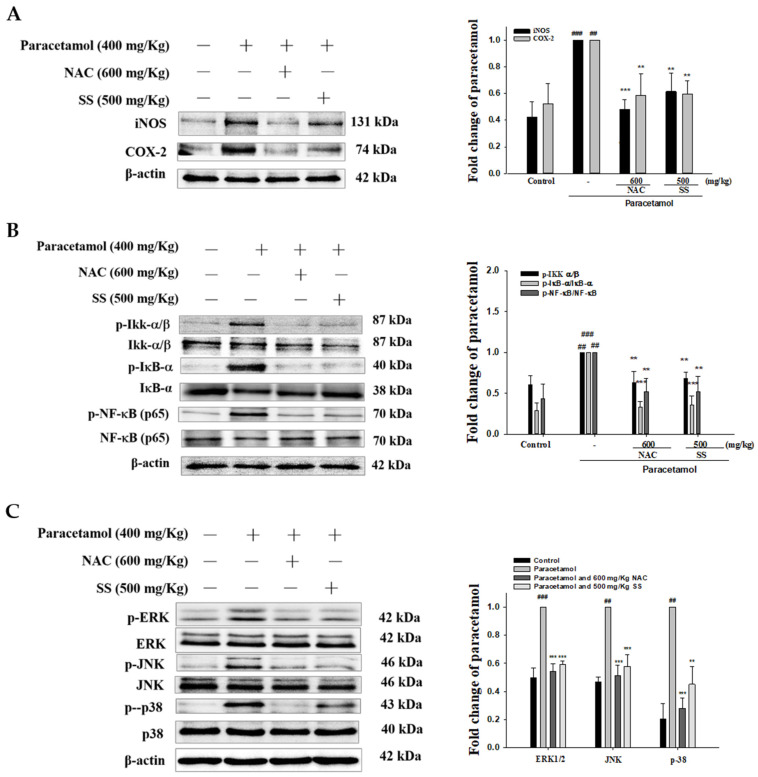
Effects of SS treatment on hepatic iNOS, COX-2 (**A**), IKK, IκBα, NF-κB (**B**), and MAPK (**C**) expression in paracetamol-induced liver injury. Total protein was extracted from liver tissues. The protein expression levels were determined by Western blotting. The bands’ intensities were quantified and normalized as per those for β-actin. The values are reported as the means ± S.E.M. of five mice per group. ^##^ *p* < 0.01, ^###^ *p* < 0.01 relative to the control group; ** *p* < 0.01, and *** *p* < 0.001 relative to the paracetamol group.

The occurrence of paracetamol-induced liver toxicity is closely related to the activation of immune responses. As depicted in Figure 3C–E and Figure 4F, the serum levels of NO, TNF-α, IL-1β, and IL-6 were increased in the paracetamol group. SS pretreatment dramatically and significantly reduced the serum levels of these proinflammatory cytokines. These results show that the hepatoprotective effects of SS may also be due to its ability to effectively inhibit inflammatory cytokines.

### 3.5. SS Inhibited Paracetamol-Induced iNOS, COX-2, and NF-κB Pathway Protein Expression

As shown in Figure 4A, the protein expression of iNOS and COX-2 was significantly increased in the paracetamol group. SS treatment significantly reduced iNOS and COX-2 levels compared with the paracetamol group. Previous studies have demonstrated that the NF-κB pathway is closely related to the production of various proinflammatory cytokines. As shown in Figure 4B, the phosphorylation of Ikk, IκBα, and NF-κB was increased by paracetamol treatment, and this was prevented by SS pretreatment. This indicates that SS might protect against paracetamol-induced inflammation by modulating the NF-κB pathway.

### 3.6. SS Inhibited Paracetamol’s Induction of MAPK Signaling Pathway

As depicted in Figure 4C, the phosphorylation of ERK, JNK, and p38 was significantly higher in the paracetamol group than the control group. Pretreatment with SS effectively decreased the hepatic expression of phosphorylated ERK, JNK, and p38 compared with the paracetamol-only group. Thus, our results suggest that SS effectively protected the liver against injury by the inactivation of the MAPK/NF-κB signaling pathway.

### 3.7. SS Relieved Oxidative Stress and Activated Protective Antioxidant Mechanisms via Keap1/Nrf2/HO-1 Signaling after Paracetamol Challenge

**Figure 5:**
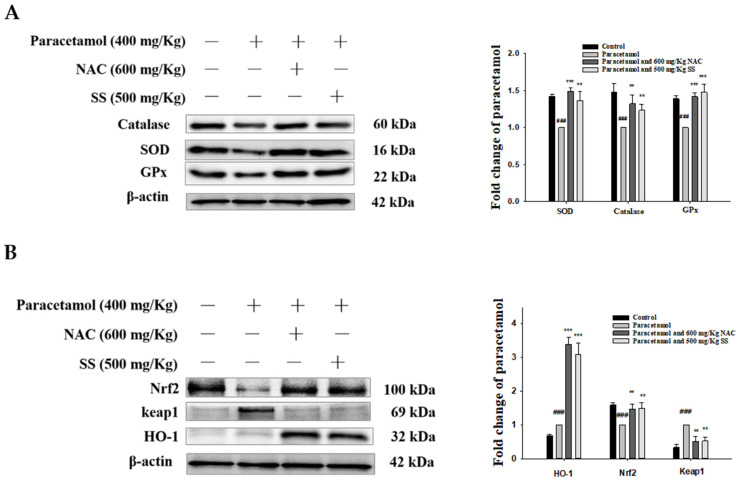
SS upregulated antioxidant enzymes (catalase, SOD, and GPx) (**A**) and activated protective mechanisms via Keap1/Nrf2/HO-1 signaling pathway (**B**) after paracetamol challenge. Total protein was extracted from liver tissues. The protein expression levels were determined by Western blotting. The bands’ intensities were quantified and normalized to those for β-actin. The values are reported as the means ± S.E.M. of five mice per group. ^###^ *p* < 0.01 relative to the control group; ** *p* < 0.01 and *** *p* < 0.001 relative to the paracetamol group.

Oxidative stress is a key factor in liver damage caused by paracetamol. The accumulation of ROS is the main cause of oxidative stress. The levels of ROS accumulation, antioxidant factors (catalase, SOD, GPx, and GSH), and oxidative stress factors (MDA) were measured to assess the state of oxidative stress induced by paracetamol. As depicted in Figure 5A, the antioxidant enzymes were significantly lower in the paracetamol group. SS improved the expression of SOD, catalase, and GPx compared to that in the paracetamol group. The results above verify the role of SS in suppressing paracetamol-induced oxidative stress in the liver. 

In order to explore the possible antioxidant mechanism of SS’s protection against stress, we evaluated the Keap1/Nrf2/HO-1 signaling pathway, which is an important antioxidant response element signaling pathway. As shown in Figure 5B, the expression of both Nrf2 and HO-1 was significantly increased by SS treatment compared to that with paracetamol only. The expression of Keap1, the main repressor of Nrf2, was significantly increased in the cytoplasm in the paracetamol-challenged animals and was reduced by SS. 

### 3.8. SS Relieved CYP2E1 Expression after Paracetamol Challenge

**Figure 6:**
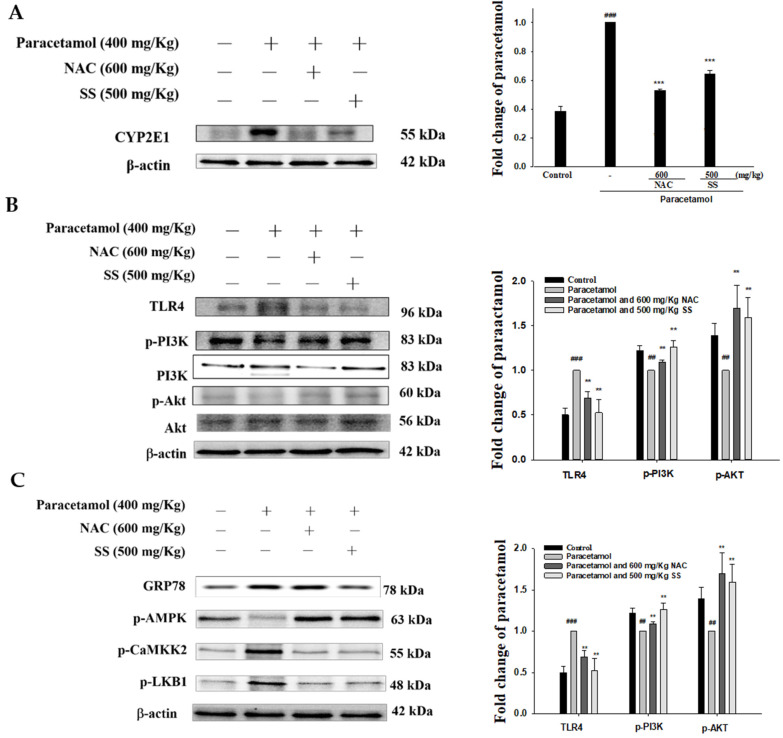
SS inhibited CYP2E1 (**A**), TLR4, PI3K, AKT (**B**), GRP78, p-AMPK, p-LKB1, and p-CaMKKβ (**C**) protein expression in paracetamol-exposed mice. Total protein was extracted from liver tissues. The protein expression levels were determined by Western blotting. The bands’ intensities were quantified and normalized to those for β-actin. The values are reported as the means ± S.E.M. of five mice per group. ^##^ *p* < 0.01, ^###^ *p* < 0.01 relative to the control group; ** *p* < 0.01 and *** *p* < 0.001 relative to the paracetamol group.

CYP2E1 is a key enzyme that causes paracetamol to be metabolized to toxic NAPQI, so we investigated whether SS affected the protein expression of CYP2E1. As depicted in Figure 6A, paracetamol injection markedly increased hepatic CYP2E1 expression. After SS treatment, CYP2E1 expression was decreased in the paracetamol-treated group. Thus, SS protected the hepatocytes against paracetamol-induced injury by suppressing CYP2E1.

### 3.9. SS Regulated TLR4/PI3K/Akt Signaling Pathway after Paracetamol Challenge

TLR4 is a key sensor that transmits inflammatory signals, which can cause the release of inflammatory mediators and initiate the migration and infiltration of inflammatory cells into diseased tissue. Thus, the TLR4/PI3K/Akt axis is closely related to cell growth and oxidative stress in the inflammatory response. As Figure 6B shows, the paracetamol-only group demonstrated an increase in the expression of TLR4 compared to the control, while SS pretreatment abrogated this increase. In addition, the phosphorylation of Akt and PI3K was decreased after paracetamol administration but increased by SS pretreatment. The results demonstrate that supplementation with SS reduced the hepatic damage by inhibiting TLR4/PI3K/Akt signaling following a paracetamol challenge.

### 3.10. SS Regulated CaMKKβ/LKB1/AMPK Signaling Pathway after Paracetamol Challenge

Endoplasmic reticulum (ER) stress can disrupt the Ca^2+^ balance in the ER, resulting in a decreased Ca^2+^ concentration and leakage into the cytoplasm. When the concentration of Ca^2+^ is increased in the cytoplasm, it activates Ca^2+^/calmodulin-dependent kinase kinase β (CaMKKβ) and AMP-activated protein kinase (AMPK), causing autophagy. Therefore, the activation of LKB1/CaMKK–AMPK signaling may damage liver tissue [29]. p-AMPK was decreased and glucose regulatory protein 78 (GRP78), p-LKB1, and p-CaMKKβ were increased after the paracetamol challenge (Figure 6C). SS treatment elevated p-AMPK and downregulated GRP78, p-LKB1, and p-CaMKKβ protein expression compared to the paracetamol-treated group. The data show that SS prevented the leakage of Ca^2+^ from the ER by regulating the CaMKKβ/LKB1/AMPK axis and blocked autophagy in the livers of paracetamol-exposed mice.

### 3.11. Blocking AMPK Synergistically with Compound C to Increase Anti-Inflammatory Capacity of SS 

**Figure 7:**
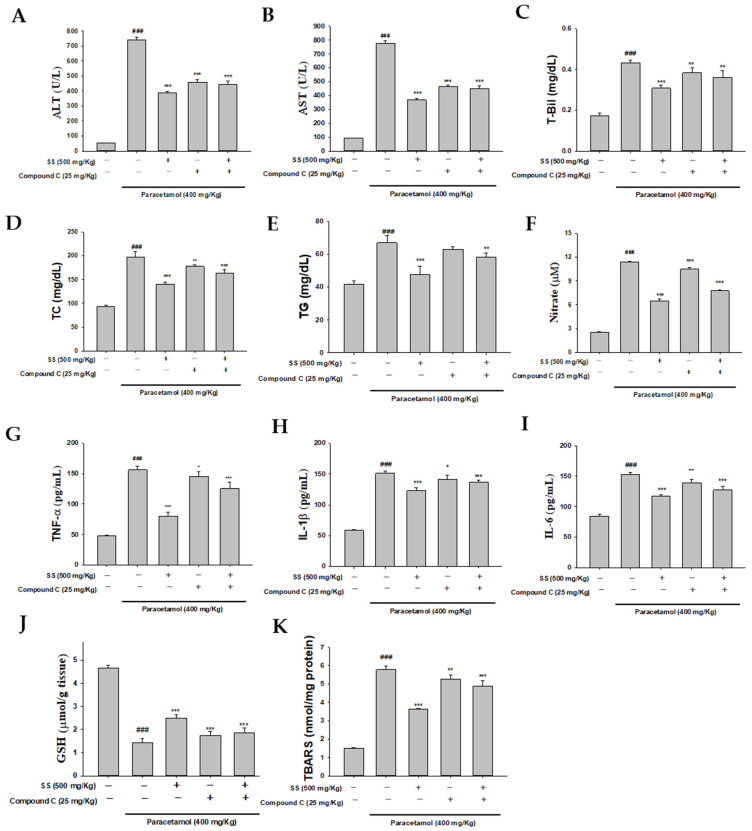
SS and AMPK inhibitor (compound C) reduced AST (**A**), ALT (**B**), T-Bil (**C**), TC (**D**), TG (**E**), NO (**F**), TNF-α (**G**), IL-1β (**H**), IL-6 (**I**), GSH (**J**), and MDA (**K**). SS was orally administered to mice for 6 days, with the last dose 1 h before paracetamol administration. The values are reported as the means ± S.E.M (*n* = 6) of five mice per group. ^###^ *p* < 0.01 relative to the control group; * *p* < 0.05, ** *p* < 0.01, and *** *p* < 0.001 relative to the paracetamol group.

In order to determine whether SS affected AMPK activity in paracetamol-triggered hepatotoxicity, we used the AMPK inhibitor compound C for further research. As depicted in Figure 7A–K, the effects of compound C were confirmed by significantly higher serum biochemical markers, lipid profiles, proinflammatory cytokine release, and levels of GSH and MDA compared to the SS-pretreatment group after paracetamol challenge. Similar results were observed for hepatic MDA. The results show that AMPK plays a key role in the protection against paracetamol-induced liver injury. In addition, the biochemical markers, lipid profiles, proinflammatory cytokine release, and levels of GSH were inhibited by co-treatment with SS and compound C compared to the paracetamol-alone group. Thus, SS may protect against paracetamol-induced acute liver failure through the CaMKKβ/LKB1/AMPK pathways. 

## 4. Discussion

Paracetamol is widely used as an analgesic and anti-fever drug globally. However, hepatotoxicity induced by an overdose of paracetamol is a common cause of acute liver failure and the main cause of drug-induced liver injury. Excessive oxidative stress, ER stress, and inflammation induced by paracetamol are the main causes of acute liver failure [30]. Although NAC is substantially effective in partially preventing paracetamol-induced hepatotoxicity, it is only effective during the early period, and some patients still show serious side effects such as nausea, vomiting, allergic reactions, and headaches [6]. The pharmacological effects of *S.* *sanghuang* have been studied by many researchers, who have characterized its antioxidant and anti-inflammatory properties. *S.* *sanghuang* is considered to be one of the most effective anti-inflammatory drugs found in higher fungi and has been widely used as a medicinal fungus. In this study, mice were orally treated with three doses of SS (125, 250, and 500 mg/kg) once daily for six consecutive days for preventing paracetamol-induced hepatotoxicity. The dosages of SS were established according to our lab’s previous paper [19,20,22]. The administration of various doses of SS did not affect the viability of murine macrophages, while the administration of a nontoxic dose of SS could significantly reduce the levels of LPS-induced NO and proinflammatory cytokines in macrophages. In addition, the protective effects of SS on inflammation induced by LPS in vitro and in vivo were mediated by suppressing the TLR4-mediated PI3K/AKT/mTOR/IKK signaling pathway [20]. Therefore, it is urgent to develop new and effective drugs for the treatment of paracetamol overdose and explore the potential molecular mechanisms.

Paracetamol overdose induces necrosis and inflammatory infiltration in the mouse liver, as well as increasing serum ALT and AST levels, indicating liver insufficiency [6,31]. In this study, the oral SS pretreatment of paracetamol-exposed mice significantly reduced the histopathological damage to the liver, including necrotic liver damage, infiltration with inflammatory cells, and hepatocyte degeneration. In addition, SS decreased the levels of serum AST, ALT, and T-Bil caused by liver functional impairment after paracetamol overdose and prevented abnormal lipid metabolism (TC and TG) in the serum. At the same time, it was found that the positive control NAC and SS have the same effect.

When liver cells are damaged, ALT and AST are released into the circulatory system [6]. In addition, after paracetamol overdose, increased levels of lipid peroxidation products have been shown to be related to mitochondrial oxidative stress and peroxynitrite formation because excessive oxidative stress triggers lipid peroxidation and leads to cell membrane destruction and cell death [32]. Thus, our data support the idea that SS can effectively protect against the liver damage caused by paracetamol, improve biochemical parameters and reduce lipid peroxidation.

Cellular GSH is essential for the detoxification of excess paracetamol because it binds to the paracetamol metabolite NAPQI, and the elimination of GSH leads to hepatocyte necrosis [33]. Our results show that excessive paracetamol can cause oxidative stress in liver tissue and hepatocyte necrosis by reducing GSH content and increasing TBARS levels and also inhibiting antioxidant enzyme activity. However, the paracetamol-induced GSH depletion, TBARS formation and reduction in antioxidant enzyme activity were significantly reversed by SS pretreatment, which might contribute to SS’ antioxidant effects. Furthermore, CYP2E1 is one of the most important indicators of drug-induced liver toxicity and liver disease [34]. In this study, after SS pretreatment, the overexpression of CYP2E1 in liver tissue caused by paracetamol exposure was reversed. Taken together, these results indicate that SS ameliorated paracetamol-induced liver damage from oxidative stress in mice.

Oxidative stress increases the expression of proinflammatory genes, and inflammatory cells subsequently similarly trigger the overproduction of ROS, resulting in a vicious circle that triggers the occurrence and development of various diseases. Thus, when exposed to various toxic substances, the liver’s detoxification mechanism plays an important role, and the inflammatory response can amplify tissue damage and lead to incorrect tissue repair [35]. Increasing evidence shows that acute paracetamol poisoning can increase the circulating levels of many proinflammatory cytokines [36]. In this study, SS pretreatment effectively reduced NO, TNF-α, IL-1β, and IL-6 secretion in paracetamol-induced acute liver failure.

The NF-κB pathway is a critical signaling axis mediating the expression of inflammation-related mediators such as via NF-κB-binding motifs in their promoters. The activation of the NF-κB protein is associated with paracetamol attack, promoting the expression of TNF-α, iNOS, and COX-2 [37]. In addition, the expression of iNOS induces the excessive production of NO, which intensifies the inflammatory response by activating inflammatory signal transduction in cells. Therefore, reducing the level of NO by inhibiting iNOS expression is considered a useful step for evaluating the efficacy of new therapies in the treatment of inflammatory diseases including paracetamol-induced hepatotoxicity [38]. Furthermore, the expression of iNOS and COX-2 proteins is related to chronic inflammatory diseases induced by oxidative stress [39]. Here, paracetamol administration led to a prominent increase in the levels of iNOS and COX-2, the phosphorylation of Ikk and IκBα, and NF-κB expression. SS pretreatment could markedly inhibit this increase. This suggests that SS could attenuate paracetamol-induced hepatic inflammation and the consequent acute liver failure.

Many studies have reported that TLR plays a crucial regulatory role in recognizing foreign pathogen-related molecular patterns (PAMPs) and damage-associated molecular patterns (DAMPs) released in oxidative stress during tissue damage [40]. Our results demonstrate that the administration of an excessive dose of paracetamol leads to increased expression of TLR4, and enhances the protein expression of MAPKs and NF-κB and the subsequent production of inflammatory mediators and pro-inflammatory factors, which ultimately leads to the development of liver failure. However, all the changes were significantly reduced by SS pretreatment. These data suggest that it is likely that SS’ suppression of paracetamol-induced inflammatory mediators and proinflammatory factor expression is mainly attributable to the inhibition of the NF-κB pathway.

Oxidative stress can further cause MAPK activation, which plays a crucial role in the intracellular signaling pathway of paracetamol-induced hepatotoxicity [41]. The MAPK family is related to cell death and is responsible for the production of ROS and proinflammatory cytokines [42]. Studies have shown that ERK is related to oxidative stress and apoptosis, and that inhibiting the ERK signaling pathway protects against paracetamol-induced hepatotoxicity by regulating proinflammatory cytokines [42]. In addition, JNK activation promotes mitochondrial dysfunction, mitochondrial oxidative stress, and ROS, leading to liver cell apoptosis when excessive paracetamol is administered. Blocking the phosphorylation of JNK can reduce liver damage in paracetamol toxicity [43]. Our Western blot data show that paracetamol activated the expression of p-ERK, p-JNK, and p-p38, leading to hepatocyte apoptosis. After the toxic effects of paracetamol, SS effectively protects the liver from damage by inhibiting the MAPK pathway.

As the main regulator protecting against oxidative stress, Nrf2 regulates the expression of antioxidant genes and phase II detoxification enzymes (such as catalase, SOD, and HO-1), which counteract oxidative stress by enhancing the removal of ROS and enhancing the antioxidant capacity of cells. In our study, paracetamol challenge led to an increased protein expression of HO-1. Compared with the paracetamol group, there was a marked increase in HO-1 protein after NAC treatment or SS pretreatment. In addition, Keap1, an inhibitor of Nrf2, acts as an adapter for the degradation of Nrf2 [44]. SS reduced the expression of the Keap1 protein in the presence of paracetamol, and this may contribute to the activation of Nrf2 induced by SS. Thus, the activation of Keap1/Nrf2/HO-1 signaling plays an essential role in inhibiting paracetamol-induced acute liver failure. Keap1/Nrf2/HO-1 signaling can control the expression of downstream antioxidant enzymes including NAD(P)H: quinone oxidoreductase 1 (NQO1) and the catalytic/modifier subunit of glutamate-cysteine ligase (GCLC/GCLM). A growing number of studies have documented that Keap1/Nrf2/HO-1 signaling mitigates oxidative stress damage by upregulating antioxidant defenses and reducing free radicals and is also an important regulator of many cytoprotective genes; it is considered a potential target for the treatment of various liver diseases. Clearly, further studies in this area focusing on the protein expression of downstream antioxidant enzymes and activity related to paracetamol metabolism are needed to completely understand these possible mechanisms.

The PI3K/AKT signaling pathway is a classic signaling pathway that plays an important role in a variety of physiological and pathological processes (such as cell survival and differentiation, cell growth, motility and apoptosis) [45]. In addition, the PI3K/AKT axis is critically modulated in TLR signaling pathways [46]. Some studies have reported that the PI3K/AKT signaling pathway is related to liver damage and early liver regeneration caused by paracetamol. The transcriptional activity of NF-κB was enhanced by the activation of the PI3K/Akt pathway [47]. Our experimental results show that SS prevented paracetamol-induced liver damage by activating the PI3K/Akt signaling pathway via protein phosphorylation.

A recent study showed that the CaMKKβ/LKB1/AMPK axis and Ca^2+^ levels could provide a quick, adaptable switch to promote the survival of cells [35]. AMPK has extensive roles in numerous pathways, especially those closely related to metabolic diseases [48]. In addition, AMPK activation prevents inflammation through the IKK/NF-κB signaling pathway [49]. CaMKKβ, an AMPK-activating kinase, may exert anti-inflammatory effects and reduce inflammatory responses to paracetamol stimulation [50]. LKB1 is a key upstream kinase and critical downstream molecule of AMPK and is essential for its activation [51]. The expression of the chaperone GRP78, an indicator of ER stress, was greatly enhanced after the downregulation of AMPK [52]. Our results further demonstrate that decreases in the phosphorylation of CaMKKβ, LBK1, and GRP78 and an increase in the phosphorylation of AMPK were induced by the treatment with SS. Furthermore, these results demonstrate that treatment with SS inhibited paracetamol-induced hepatotoxicity via upregulation of the CaMKKβ/LKB1/AMPK signaling pathway.

AMPK activation can alleviate pathologies related to oxidative stress by improving redox balance, autophagy flux, and nicotinamide adenine dinucleotide homeostasis [53]. Recent studies showed that compound C downregulated p-AMPK and promoted paracetamol-induced hepatotoxicity in hepatocytes [54]. Therefore, we used compound C to test our concept. The results show that treatment with compound C aggravated paracetamol-induced hepatotoxicity in mice by inactivating AMPK. In addition, as expected, the AMPK-inhibitory effect induced by compound C abolished the protective effect of SS on paracetamol-induced hepatotoxicity, and increased biochemical markers, the lipid profiles, proinflammatory cytokines, and the levels of GSH after paracetamol challenge. Collectively, compound C regulated the phosphorylation of AMPK, and SS’ hepatoprotective effects on paracetamol-induced hepatotoxicity might be, at least in part, mediated by modulating the CaMKKβ/LKB1/AMPK signaling pathway.

## 5. Conclusions

**Figure 8:**
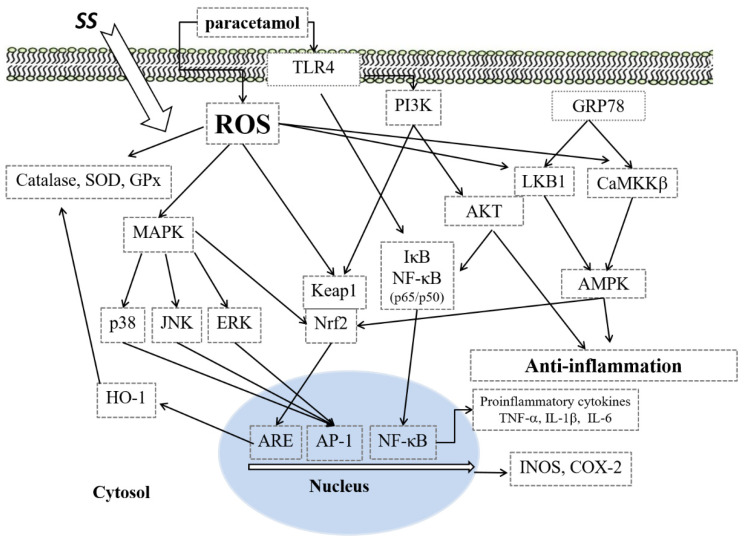
The mechanism for the protective effect of SS on paracetamol-induced inflammation.

In this study, we provided novel evidence that SS displays significant therapeutic efficacy against paracetamol-induced hepatotoxicity by suppressing oxidative stress and the inflammatory response in mice. The mechanisms of action were revealed to involve SS’ potent antioxidant and anti-inflammatory properties, mediated by inhibiting the protein expression of the proinflammatory mediators iNOS and COX-2; suppressing the NF-κB and MAPK signaling pathways; modulating the Keap1/Nrf2/HO-1, TLR4/PI3K/Akt, and CaMKKβ/LKB1/AMPK signaling pathways; and suppressing oxidative stress (Figure 8). Therefore, the extract of the mycelium of SS has potential in the prevention of inflammation-related diseases, such as paracetamol-induced hepatotoxicity.

## Data Availability

The data presented in this study are available on request from the corresponding author.
